# An asymmetry of treatment between lotteries involving gains and losses in rhesus monkeys

**DOI:** 10.1038/s41598-019-46975-2

**Published:** 2019-07-18

**Authors:** Aurélien Nioche, Sacha Bourgeois-Gironde, Thomas Boraud

**Affiliations:** 1 Aalto University, School of Electrical Engineering, Department of Communications and Networking, 02150 Espoo, Finland; 2 Institut Jean Nicod, Département d’études cognitives,ENS, EHESS, PSL Research University, 75005 Paris, France; 3 Institut Jean Nicod, Département d’études cognitives, CNRS, UMR 8129, Paris, France; 4 Institut des Maladies Neurodégénératives, Université de Bordeaux, 33000 Bordeaux, France; 5 Institut des Maladies Neurodégénératives, CNRS, UMR 5293, Bordeaux, France; 6 Laboratoire d’Economie Mathématique et de Microéconomie Appliquée, Université Panthéon Assas, 75006 Paris, France; 7 Centre Expert Parkinson, CHU Bordeaux, 33000 Bordeaux, France

**Keywords:** Computational neuroscience, Animal behaviour

## Abstract

Decision-making in humans is known to be subject to several biases. For instance, when facing bets, humans demonstrate some asymmetry concerning their preference for the riskiest option depending on whether stakes involve potential gains or potential losses. They are indeed risk-averse for bets involving gains but risk seeking for bets involving losses. They also exhibit a distorted perception of probabilities. It is not clear whether non-human primates exhibit the same biases. Setting up a protocol that allowed two rhesus monkeys to make choices between lotteries involving either gains or losses, we demonstrated that rhesus monkeys facing bets exhibited an asymmetry in the treatment of gains and losses comparable with that of humans.

## Introduction

**Figure 1:**
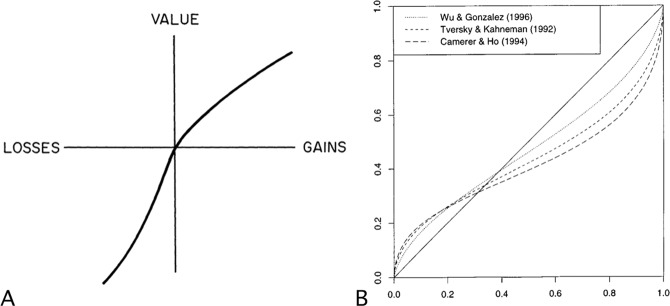
Attitude toward risk: gain-loss asymmetry and probability distortion. (**A**) A typical utility function for a human subject. The concavity of the curve for gains indicates risk-aversion, while the convexity of the curve for losses indicates risk seeking. Loss aversion is indicated by a steeper curve for losses than for gains (figure reproduced from Tversky & Kahneman^36^). (**B**) The function of distortion probabilities as they have been described by Tversky & Kahneman^4^, Camerer & Ho^37^ and Wu & Gonzalez^38^. All these functions have in common that small probabilities are over-weighted while high probabilities are under-weighted (figure reproduced from Gonzalez & Wu^19^).

Attitudes toward risk include different behavioral features that can be studied independently: risk-aversion, loss aversion, and the distortion of probabilistic information (see Fig. 1). The connection between risk-aversion and the utility of action for an individual was established early in the history of decision-theory^1,2^. Loss aversion and distortion of probabilities have been identified much more recently when expectations of gains and losses were studied from the current reference point of the decision maker^3,4^. Indeed, the seminal studies from Tversky and Kahneman show that humans (i) are generally risk-averse for bets involving gains but risk seeking for bets involving losses, (ii) exhibit loss aversion, and (iii) their perception of probabilities is distorted in the sense that small probabilities are over-weighted and high probabilities are under-weighted. These works led to the formulation of the cumulative prospect theory—a common denomination of the second version of the model of Kahneman and Tversky^5^—that made the psychological insights compatible with the maximization of a utility function^4^.

In view of the phylogenetic proximity between monkeys and apes (that include human), it seems of interest to study attitude toward risk in the former in order to extend the descriptive validity beyond the realm of human decision making. Oddly enough, and despite the fact that risk-aversion has been reported in many other species, including bees^6^ and peas^7^, several studies in rhesus monkeys (*Macaca mulatta*) report preferences for the riskier option over the safer one when the expected values are equivalent^8–12^. It is also noticeable that such preference has also been documented in chimpanzees^13^. However, there are also some studies that report risk-aversion for gains in rhesus monkeys^14^. In order to explain these discrepancies, some authors have advanced the idea that confounding factors could skew the data in the studies reporting risk seeking^15,16^.

Nonetheless, most of the previous studies focus on the attitude toward risk for gains only and leave open the question to know if it exists some asymmetry of treatment when dealing with gains compared to when dealing losses, one aspect which is central in the prospect theory^3,4^.

The objective of the present study is thus to evaluate whether the asymmetry of treatment between gains and losses seen in humans can also be evidenced in non-human primates, and if so, provide a fine description of this asymmetry by characterizing it toward (i) risk-aversion, (ii), probability distortion, (iii) stochasticity in choice.

For this purpose, we designed an experimental paradigm in which rhesus monkeys had to choose between options involving either gains or losses in reference to some initial stock at the beginning of each trial. Let us highlight here that we assessed losses in a reference-dependent way. No “real” losses have been experienced by the monkeys such that, when their outcome fell below the reference point, they could still receive a reward. It was less than what they could expect, hence only a loss relative to a reference point. In other studies that have studied loss aversion with human or non-human primates, “loss” is, as far as we know, understood in this particular relative sense, not in an absolute one^17,18^.

We fitted their behavioral data to a cumulative-prospect-theory based decision model to assess the contrast between the treatments of losses and gains.

## Results

### Task

We trained two monkeys, Monkey H and Monkey G, to perform choices when facing a pair of lotteries. At each trial, the monkey had to touch one of two circular targets on a touchscreen, each of them associated with a lottery. Each lottery *L*_*i*_ had two outcomes, a positive or negative amount *x*_*i*_ and 0. The amount *x*_*i*_ is the actual outcome of the lottery with probability *p*_*i*_, and 0 is the actual outcome with probability 1 − *p*_*i*_. For example, a trial could offer the possibility of choosing between (1) half of chance to get 2 tokens (*p*_1_ = 0.5, *x*_1_ = 2), and (2) one chance over four to get 3 tokens (*p*_2_ = 0.25, *x*_2_ = 3).

**Figure 2:**
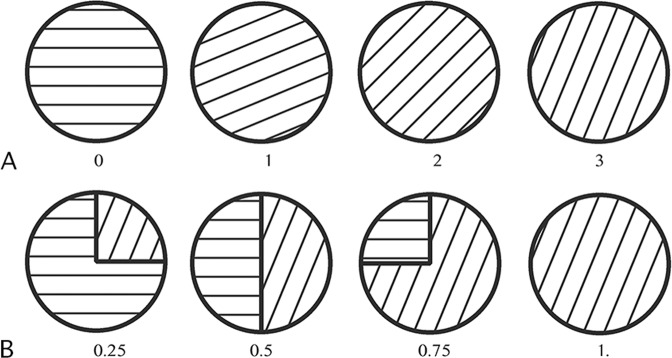
Representation of outcomes and probabilities. (**A**) Quantity of token earned (lost) is encoded by the pattern. From left to right: 0, 1, 2 and 3 tokens (rotation of the horizontal lines in the other direction would indicate a negative amount). (**B**) Each lottery with non-degenerate probability is represented by a pie chart composed of two slices. The arc length of each slice represents the probability associated to the amount corresponding to the pattern of this slice. From left to right: 3 token gain with probability 0.25, 0.5, 0.75 and 1.

**Figure 3:**
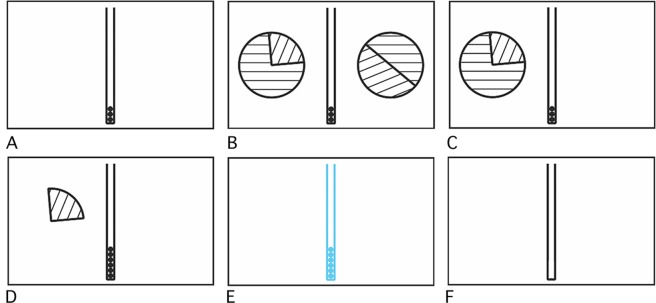
Experimental paradigm. (**A**) The monkey has to grasp a grip (50~300 ms). (**B**) Stimuli appear. In this example, on the left side: 3 tokens with probability 0.25 (0 otherwise); on the right side: 1 token with probability 0.5 (0 otherwise). (**C**) Once the choice is made, only the lottery chosen remains. (**D**) After returning the hand to the grip, displaying of the outcome and addition (removing) of the tokens from the gauge. (**E**) End of the trial: gauge draining and admission of the reward. (**F**) Inter-trial.

Amounts were represented by texture (different line orientations) and probabilities by the slice taken by the corresponding outcome on the target (see Fig. 2). For implementing losses, the monkey was rewarded at the end of the trial with an amount of water proportional to the level of gauge filled with tokens. Starting each trial with 3 tokens, the monkey could win or lose up to 3 tokens (see Fig. 3).

The lottery pairs can be characterized according to two criteria: (i) Do the pair contain lotteries with potential gains, or potential losses, or both? (ii) Do the pair contain a lottery that is a stochastic dominant option (i.e. a lottery *A* such as *p*_*A*_ > *p*_*B*_ and *x*_*A*_ > *x*_*B*_)? For commodity purpose, we will name thereafter lottery pairs with potential gains only LPG, and lottery pairs with potential losses only LPL.

Therefore, we distinguished 7 types of lottery pairs:

- **Type 1**. *x*_1_ > 0 and *x*_2_ < 0 while *p*_1_ = *p*_2_ (lottery pairs containing one lottery with potential losses and on lottery with potential gains); assess the discrimination of *losses from the gains*; 36 different lottery pairs.
- **Type 2**. *p*_1_ = *p*_2_ and *x*_1_ > *x*_2_, with *x*_*i*∈1,2_ > 0 (LPG with a stochastic dominant option differentiating only by the *x* values); assess the discrimination of *positive x*-values; 12 different lottery pairs.
- **Type 3**. *p*_1_ = *p*_2_ and *x*_1_ < *x*_2_, with *x*_*i*∈1,2_ < 0 (LPL with a stochastic dominant option differentiating only by the *x* values); assess the discrimination of *negative x*-values; 12 different lottery pairs.
- **Type 4**. *p*_1_ > *p*_2_ and *x*_1_ = *x*_2_, with *x*_*i*∈1,2_ > 0 (LPG with a stochastic dominant option differentiating only by the *p* values); assess the discrimination of *p*-values associated to *positive x*-values; 12 different lottery pairs.
- **Type 5**. *p*_1_ < *p*_2_ and *x*_1_ = *x*_2_, with *x*_*i*∈1,2_ < 0 (LPL with a stochastic dominant option differentiating only by the *p* values); assess the discrimination of *probabilities* associated to *negative* quantities; 18 different lottery pairs.
- **Type 6**. *p*_1_ < *p*_2_ and *x*_1_ > *x*_2_, with *x*_*i*∈1,2_ > 0 (LPG with *no* stochastic dominant option); 18 different lottery pairs.
- **Type 7**. *p*_1_ < *p*_2_ and *x*_1_ < *x*_2_, with *x*_*i*∈1,2_ < 0 (LPL with *no* stochastic dominant option); 18 different lottery pairs.

Type 1–5 lottery pairs have been used for assessing performance, while Type 6 and 7 have been used for assessing attitude toward risk. Indeed, for these two last types, the monkey had to realize trade-offs between quantity and probability: either he can have a greater quantity but with a smaller probability (the riskiest option), or he can have a smaller quantity but with a greater probability (the safest option).

### Certainty-risk trade-off

In this first section, we report results for the case similar to that proposed by Kahneman and Tversky^3^. The two monkeys were faced with two lottery pairs. In the first, they had the choice between a lottery giving one token with certainty and one giving two tokens half of the time. The second pair was similar to the first, except that the amounts were negative.

**Figure 4:**
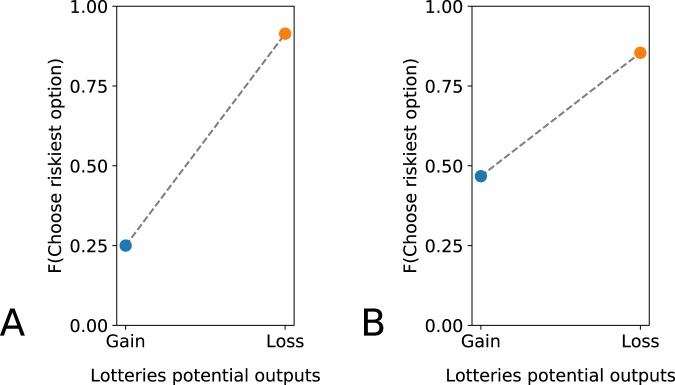
Certainty-risk trade-off. Each dot corresponds to the frequency with which the riskiest lottery was chosen, when the choice was between two lotteries with equal expected values but one of the both having a certain outcome. Blue: potential gains only; orange: potential losses only. (**A**) Monkey H. (**B**) Monkey G.

Figure 4 shows the results for these two lottery pairs (Monkey H: 251 trials, Monkey G: 236 trials). The monkeys behaved similarly to the behavior reported in humans in similar trade-offs^3^: the monkeys preferred the safest option if the lottery offered a gain and the riskiest option if the lottery offered a loss (Monkey H: *dll* = 1, *χ*^2^ = 301.39, *p* < 0.01; Monkey G: *dll* = 1, *χ*^2^ = 89.37, *p* < 0.01).

### Respect of first-order stochastic dominance

In order to assess accurately the performance of the task by the two monkeys, we consider separately the lottery pairs where there was a strictly dominant option (lottery pairs Type 1–5).

**Figure 5:**
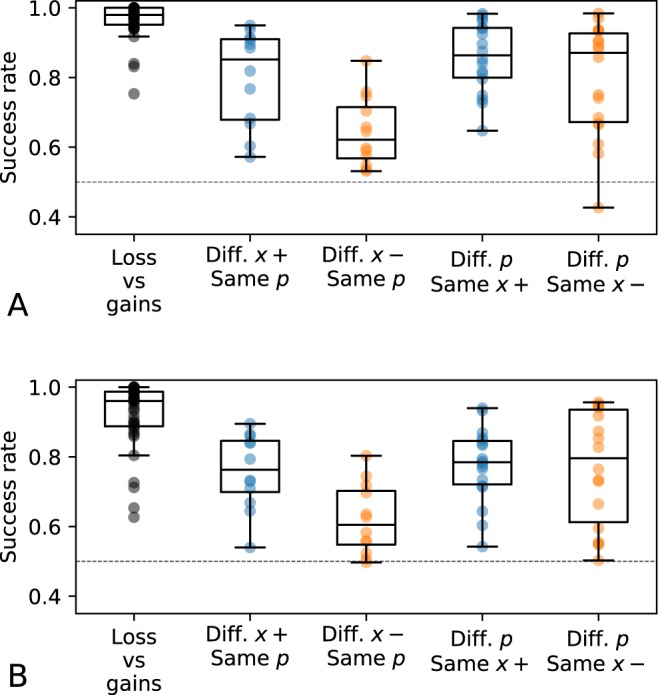
Performance assessing. Only lottery pairs with a dominant option are considered. Each dot represents the frequency with which the best option was chosen for a particular pair of lotteries. Blue: lottery pairs with potential gains only; orange: lottery pairs with potential losses only. Each box plot extends from the lower to the upper quartile of frequencies observed, with the central line at the median. The whiskers represent the value of 1.5 IQR. As they are two options, chance level, indicated by a dashed line, is at 0.5. (**A**) Monkey H. (**B**) Monkey G.

The results of the 96 lottery pairs meeting this condition are summarized in Fig. 5 (Monkey H: 19594 trials; Monkey G: 16938 trials). In the supplementary section, Table S3 provides the median success rate for each type of lottery pairs and Fig. S1 provides a similar representation of performances as Fig. 5, but considering separately 20 chunks of data in order to control for the stability of performances over time.

As the task design includes only two choice options, the chance level is 0.5. Results show that all interquartile ranges of success rates are above 0.5 (min: 0.61, max: 0.98), indicating that monkeys performed better than random for Type 1–5 lottery pairs. However, both monkeys performed worst in the condition where they had to choose between two lotteries that had two different potential losses with the same probability.

### Consideration of difference of expected values during trade-offs between quantity and probability

Using the results for lottery pairs where it is possible to distinguish a safer option and a riskier option (lottery pairs Type 6 & 7), we checked that the frequency with which the riskiest option was chosen was dependent on the difference between the expected values of the safest option and the riskiest option. To assess this relationship we used an ordinary sigmoid function with two free parameters, one for the slope (*β*) and one for the intercept (*γ*), that we optimized to fit the data. This function is supposed to increase monotonically (i.e. *β* > 0): the more the difference between expected values favors the riskiest option, the more the monkey is expected to choose this option. As we fitted separately the data obtained with LPG, and those obtained with LPL, we optimized in all four parameters: *β*_*G*_, *β*_*L*_, *γ*_*G*_, *γ*_*L*_.

**Figure 6:**
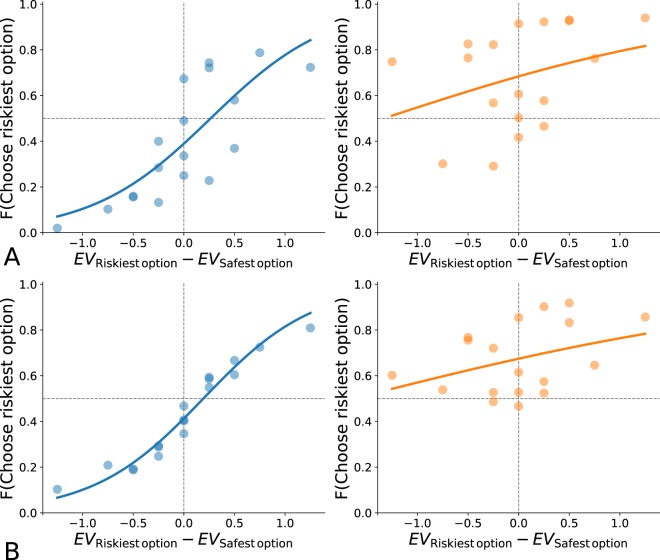
Frequency of risky choice over differences of expected value. Each dot represents the frequency with which the riskiest option is chosen for a particular pair of lotteries with no stochastic dominance. (**A**) Monkey H. Left: lottery pairs without stochastic dominant option involving gains (blue). Right: lottery pairs without stochastic dominant option involving losses (orange). (**B**) Monkey G. Left: lottery pairs without stochastic dominant option involving gains (blue). Right: lottery pairs without stochastic dominant option involving losses (orange).

Data used were obtained on 18 LPG and 18 LPL without stochastic dominant option (Monkey H: 4952 trials, Monkey G: 4435 trials). Results are shown in Fig. 6. In the supplementary section, Table S4 summarizes the results of the optimization process.

The slope of the curve of the sigmoid function in Fig. 6A,B on the left side (in blue) is steep and the points are close to the curve, indicating that both monkeys consider the difference of expected values when realizing trade-offs between quantity and probability for lotteries with potential gains (Monkey H: *β*_*G*_ = 1.70 [0.72, 2.68], Monkey G: *β*_*G*_ = 1.83 [1.55, 2.12]).

However, the slope of the curve of Fig. 6A,B on the right side (in orange) is smoother and the points are further from the curve than for gains. This indicates that both monkeys less consider the difference of expected values when dealing with lotteries including potential losses, and we can not reject the hypothesis that they do not consider it at all (Monkey H: *β*_*L*_ = 0.58 [−0.36, 1.52], Monkey G: *β*_*L*_ = 0.45 [−0.17, 1.07]).

### Attitude toward risk in non-human primates

Using the results for lottery pairs where it is possible to distinguish a safer option and a riskier option (lottery pairs Type 6 & 7), we fitted a decision-making model to better identify how the macaques behave in relation to risk.

This model includes six free parameters:

• *ω*_*G*_ and *ω*_*L*_ that describe to which extent the monkey is risk-averse for lottery pairs involving respectively gains and losses. Reference values are −1 (extremely risk-seeking), 0 (risk-neutral), and 1 (extremely risk-averse).
• *α*_*G*_ and *α*_*L*_ that describe to which extent the monkey has a distorted perception of probabilities for lottery pairs involving respectively gains and losses. Reference values are 0 (maximal distortion) and 1 (no distortion).
• *λ*_*G*_ and *λ*_*L*_ that describe to which extent the decision-making process is stochastic for lottery pairs involving respectively gains and losses. Reference values are 0 (maximal stochasticity) and ∞ (no stochasticity at all).

We optimized these free parameters for each monkey separately (Monkey H: 4952 trials, Monkey G: 4435 trials). In order to control for the stability over time of the fit, we separated for each monkey the dataset in 20 chunks with respect to the chronological order of the trials, obtaining 20 best-fit parameter values for each parameter.

For describing to which extent the two monkeys present asymmetry of treatment between gain and loss, we compared the best parameters values contrasting those computed after the fitting of results obtained with 18 LPG without stochastic dominant option, and those computed after the fitting of results obtained with 18 LPL whitout stochastic dominant option.

We assessed the statistical relevance of the comparisons with the Mann-Whitney’s U ranking test, applying the Bonferroni correction for multiple comparisons.

**Figure 7:**
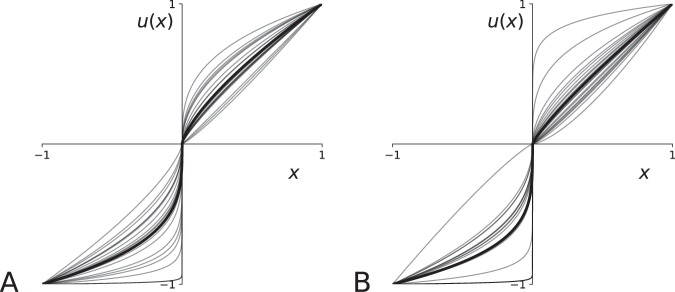
Utility functions. Built upon data fitting, they are characterized by the optimization of two parameters: *ω*_*G*_, describing to which extent the monkey is risk-averse for gains, *ω*_*L*_ describing to which extent the monkey is risk-averse for losses. To assess the stability of time, we compute the best parameter values over 20 chunks of trials (cross-validation). The thin lines correspond to the result of the fitting of these 20 chunks and the bold line correspond to the curve of the function using as parameter value the average value of the best values. (**A**) Monkey H. Data fitting reveals a slight risk-aversion for gains contrasting with a strong risk-aversion for losses (*ω*_*G*_ = 0.28 ± 0.21, *ω*_*L*_ = −0.71 ± 0.20; *u* = 0.0, *p* < 0.001, *n*_*obs*_ = 2 × 20). (**B**) Monkey G. Data fitting reveals a quasi risk neutrality for gains contrasting with a strong risk-aversion for losses (*ω*_*G*_ = 0.15 ± 0.31, *ω*_*L*_ = −0.75 ± 0.28; *u* = 11.0, *p* < 0.001, *n*_*obs*_ = 2 × 20).

**Figure 8:**
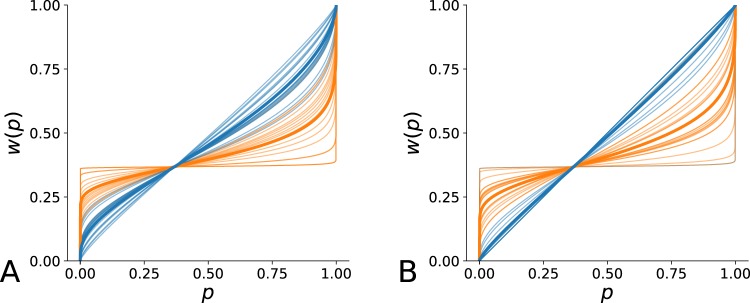
Probability distortion functions. For each monkey, two probability distortion functions are drawn: one for lottery pairs involving gains (in blue), one for lottery pairs involving losses (in orange). Based on data fitting, each of these functions is characterized by the optimization of one parameter, respectively *α*_*G*_ and *α*_*L*_. These parameters describe to which the probabilities are distorted. To assess the stability of time, we compute the best parameter values over 20 chunks of trials (cross-validation). The thin lines correspond to the result of the fitting of these 20 chunks and the bold line correspond to the curve of the function using as parameter value the average value of the best values. (**A**) Monkey H. Data fitting reveals a slight probability distortion for gains contrasting with a strong probability distortion for losses (*α*_*G*_ = 0.63 ± 0.14, *α*_*L*_ = 0.21 ± 0.14; *u* = 7.0, *p* < 0.001, *n*_*obs*_ = 2 × 20). (**B**) Monkey G. Data fitting reveals a quasi linear distortion of probabilities for gains contrasting with a strong probability distortion for losses (*α*_*G*_ = 0.90 ± 0.23, *α*_*L*_ = 0.30 ± 0.21; *u* = 24.5, *p* < 0.001, *n*_*obs*_ = 2 × 20).

**Figure 9:**
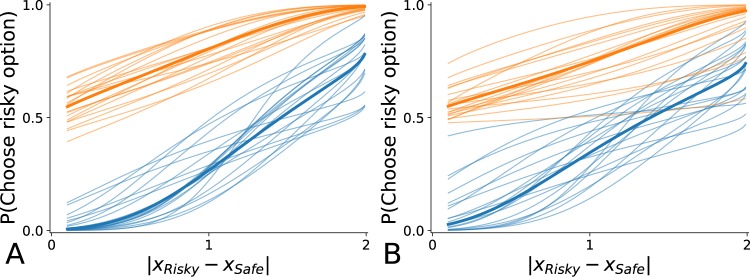
Stochasticity functions. For each monkey, two stochasticity functions are drawn: one for lottery pairs involving gains (in blue), one for lottery pairs involving losses (in orange). Based on data fitting, each of these functions is characterized by the optimization of one parameter, respectively *λ*_*G*_ and *λ*_*L*_. These parameters describe to which extent the decision-making is stochastic (the closer *λ* is to zero, the more the decision-making process is stochastic). In blue is depicted how the probability of choosing a risky lottery that gives 3.00 with probability 0.25 increases as the amount with certainty of a second lottery decreases progressively from 2.99 to 0.10. In orange is depicted how the probability of choosing a risky lottery that results in a loss of −1.00 with probability 0.25 increases as the loss with certainty resulting from a second lottery increases progressively from −0.10 to 2.99. The smoother the slope, the more the choice is stochastic (the difference of curve height is not relevant for assessing stochasticity). To assess the stability of time, we compute the best parameter values over 20 chunks of trials (cross-validation). The thin lines correspond to the result of the fitting of these 20 chunks and the bold line correspond to the curve of the function using as parameter value the average value of the best values. (**A**) Monkey H. Data fitting reveals no significant difference in stochasticity of choice for gains and losses (*λ*_*G*_ = 2.52 ± 0.98, *λ*_*L*_ = 1.82 ± 0.47; *u* = 104.0, *p* = 0.029, *n*_*obs*_ = 2 × 20). (**B**) Monkey G. Data fitting reveals no significant difference in stochasticity of choice for gains and losses (*λ*_*G*_ = 1.71 ± 0.95, *λ*_*L*_ = 1.28 ± 0.66; *u* = 153.0, *p* = 0.625, *n*_*obs*_ = 2 × 20).

A visual representation of the functions for which these parameters are used is provided in Figs 7–9. In the supplementary section, Table S5 summarizes the results of the optimization process, Table S6 provides a summary of the statistical tests, Fig. S2 provides a visual representation of parameter values over time, and Table S7 summarizes the results of a regression analysis of the evolution of the best-fit parameters values (the only statistically relevant result concerns the evolution of *ω*_*L*_ parameter for Monkey H, *β* = −0.02, *F* = 16.315, *p* = 0.009, *n*_*obs*_ = 2 × 20, showing that Monkey H is slightly more risk-averse for LPL over time).

The optimized values of *ω*_*G*_ and *ω*_*L*_ allow reconstructing the utility functions of the two monkeys, as shown in Fig. 7. For both monkeys, these values indicate a slight risk-seeking for gains or quasi risk neutrality contrasting with a strong risk-aversion for losses (Monkey H: *ω*_*G*_ = 0.28 ± 0.21, *ω*_*L*_ = −0.71 ± 0.20; *u* = 0.0, *p* < 0.001, *n*_*obs*_ = 2 × 20; Monkey G: *ω*_*G*_ = 0.15 ± 0.31, *ω*_*L*_ = −0.75 ± 0.28); *u* = 11.0, *p* < 0.001, *n*_*obs*_ = 2 × 20).

The optimized values of *α*_*G*_ and *α*_*L*_ allow reconstructing the probability distortion functions of the two monkeys, as shown in Fig. 8. These values indicate a slight probability distortion for gains contrasting with a strong probability distortion for losses (Monkey H: *α*_*G*_ = 0.63 (±0.14, *α*_*L*_ = 0.21 ± 0.14; *u* = 7.0, *p* < 0.001, *n*_*obs*_ = 2 × 20; Monkey G: *α*_*G*_ = 0.90 ± 0.23), *α*_*L*_ = 0.30 ± 0.21); *u* = 24.5, *p* < 0.001, *n*_*obs*_ = 2 × 20).

The optimized values of *λ*_*G*_ and *λ*_*L*_ allow reconstructing the stochasticity functions of the two monkeys, as shown in Fig. 9. The comparison of these values for each monkey reveals no statically difference in stochasticity of choice for gains and losses (Monkey H: *λ*_*G*_ = 2.52 ± 0.98, *λ*_*L*_ = 1.82 ± 0.47; *u* = 104.0, *p* = 0.029, *n*_*obs*_ = 2 × 20; Monkey G: *λ*_*G*_ = 1.71 ± 0.95, *λ*_*L*_ = 1.28 ± 0.66; *u* = 153.0, *p* = 0.625, *n*_*obs*_ = 2 × 20).

## Discussion

This study has developed a new paradigm assessing attitude toward risk in macaque monkeys facing lotteries that comprised potential gains and potential losses. Both macaques investigated displayed an asymmetric behavior toward gain and loss. They were slightly risk-averse or risk neutral for gains but strongly risk seeking for losses. They also exhibited either a slight probability distortion or a linear perception of probabilities for gains but a strong probability distortion for losses. Such asymmetry of treatment between lotteries involving gains and losses is congruent with the predictions of the prospect theory and is similar to what has been observed in humans^3,4^. However, the fact that we observe a slight risk-aversion for gains constitutes a discrepancy with the results of several studies in monkeys^11,18^, which leads to several questions.

### Respect of first-order stochastic dominance

Before considering these questions, let us highlight that despite a large number of different lottery pairs, both monkeys generally demonstrate good performance on lotteries pairs with stochastic dominance.

The large number of choice situations rules out the possibility that the monkeys would have been able to memorize the best choice in each case, without decoding the stimuli (the protocol included 126 different pairs of lotteries, with stimuli rotated randomly from one trial to another).

However, in the lottery pairs with a stochastic dominant option where probabilities were fixed and magnitudes varied (see Fig. 5), they were less accurate (i.e. success rate was only about 60%). Since the monkeys had still good performances when choosing between lotteries containing similar losses but with different probabilities (i.e. they selected more than 80% of the time the lottery with the smallest probability of loss), it could indicate a difficulty to discriminate the magnitude of losses.

### Consideration of difference of expected values when realizing trade-offs between quantity and probability

Our data show that both monkeys put more consideration to the difference of expected values between the riskiest and the safest option to make their choice for lottery pairs without stochastic dominant option involving gains, than to make their choice when losses were involved. This result is to put in relation to the one obtained in lottery pairs with a stochastic dominant option that show that they may have some difficulty to discriminate the magnitude of losses. Indeed, as expected value integrate not only probability but also quantity, assessing with difficulty the magnitude of losses could prevent the consideration of expected values when realizing trade-off between quantity of probability.

### Stochasticity in choice

Our data show that the stochasticity in choice is not significantly different for lottery pairs involving losses than for those involving gains, as captured by the precision parameter in our data fitting.

### Probability distortion

A strong probability distortion has been observed in humans^19^ and rhesus monkeys^11,18^ for lotteries involving gains and was therefore expected in this study. However, we observe only a moderate one in lotteries involving gains. Though, we observe a strong probability distortion for lotteries involving losses.

Unfortunately, to the extent of our knowledge, the previous studies on probability distortion either did not mixed losses and gains^11^ or did not use a different parametrization for both^18^, which makes the results difficult to compare. Therefore, even if such asymmetry of probability perception is not predicted by the prospect theory^3,4^, it would be interesting to know if such asymmetry in probability distortion and can be generalized to other species, including humans.

### Asymmetric attitude toward risk

In this study, we observed a strong asymmetry in attitude toward risk between gains and losses for both monkeys. Both monkeys exhibited a slight risk-aversion or risk neutrality for gains while exhibiting a strong risk-seeking behavior for losses.

If our results fulfill the predictions of the prospect theory^3,4^ to that respect, they do not replicate the previous results in rhesus monkeys for what concerns attitude toward risk in gains. Indeed, several studies report a tendency to risk-seeking for gains^8,10,12,18^. We can consider several explanations. (i) In previous studies, the amount of reward is generally low (the stake for a bet is generally lying between 100 and 300 of water^16^) and this could create an incentive to prefer risky options, effect that has been labeled as the *peanuts effect*^20^. In our task, at the start of each trial, the monkeys had an initial amount of water, to which their gains (losses) were added (subtracted). This makes the stake for a single trial larger, maybe allowing the *peanuts effect*^20^ to be avoided. However, this could hardly be the only factor as it had been show that risk-seeking in rhesus monkeys were robust to the manipulation of the magnitude of stakes^21^. (ii) The number of different pairs of lottery was often lower in the previous studies, their findings could rather be specific to the lottery pairs chosen. However, a risk-seeking behavior has been also obtained with probabilities of outcomes drawn from a continuous distribution^22^, what seems to rule out this possibility. (iii) A similar bet is proposed several times in the same session. This could lead to a bias, as it had been shown in human that repetition of gambles can reduce risk-aversion^23^. (iv) In a recent study that implement also losses^18^, the introduction of a small amount of water for each successful trial is maybe the cause of the report of risk seeking for gains: the certainty for the monkeys to have a small reward may induce an incentive to choose the riskiest option. (v) Strong individual differences have been noted in humans and can also be expected to occur in rhesus monkeys (reason why we analyzed the behavior of the two monkeys separately). (vi) An another possibility is that this discrepancy is due to what is called the *description-experience gap*^24^. For instance, Heilbronner and Hayden^25^ show that rhesus monkeys were less risk-seeking when reward contingencies were described than when they were experienced. More generally, the way of presentation of stimuli has been shown to greatly impact on decision-making processes in humans and monkeys^26^, and it had been shown since several decades that risk attitude is sensitive to the design of the task that aims to assess it, in humans^23^ but also in non-human species as birds^27^.

Concerning the attitude toward losses, our data are consistent with the two studies that addressed specifically the question and revealed a risk-seeking behavior for losses in capuchins^17^, or rhesus monkeys^18^. The latter indeed proposed an experimental paradigm close to ours that mixes losses and gains and found risk-seeking behavior both for gain (see above) and losses.

One aspect that deserves to be highlighted is that if monkeys seemed to seek risk when dealing with potential losses, this behavior is combined with difficulty to evaluate the quantity of loss (see Fig. 5). It is thus hard to discriminate if the animals do seek for risk or just try to avoid losses whatever the magnitude. This could be assimilated to an adaptive behavior in the sense that if an individual cannot correctly evaluate the amount of a loss, one solution is to avoid all loss as far as possible, leading to adopt a seemingly risky behavior. This could indeed explain why our two monkeys are far more risk-seeking when facing potential losses than they are risk-averse when facing potential gains.

Even if more data would be needed to explore the validity of such explanation, the present study has demonstrated that we can retrieve in rhesus monkeys an asymmetric behavior toward gain and loss similar to that reported in humans and described by the prospect theory^3,4^. This brings an argument in favour of the use of the macaque model for the study of decision making under risk and the investigation the underlying neural mechanisms. Although, the discrepancy of the results concerning attitude toward risk in rhesus monkeys appeals to conduct further studies in order to better understand how the context in which the assessment of such attitude is done impacts on the results obtained.

## Materials and Methods

### Behavioral protocol

#### Animals

The study was performed on two female rhesus monkeys, Monkey H, 3-year-old at the time of the experiment (born in 2012), and Monkey G, 4-year-old at the time of the experiment (born in 2011). The monkeys were housed in the animal facility of the Institute of Neurodegenerative Diseases (UMR CNRS 5293, Bordeaux, France) under standard conditions (a 12 h light/dark cycle with the light on from 7.00 am to 7.00 pm; humidity at 60%, temperature 22 °C ± 2 °C). Experimental procedures were performed in accordance with the Council Directive of 2010 (2010/63/EU) of the European Community and the National Institute of Health Guide for the Care and Use of Laboratory Animals. The protocol received agreement from the Ethical Committee for Animal Research CE50 (agreement number: C33063268). Data used for analysis were collected over 151 days (Monkey H: 195.07 ± 102.42 SD trials per day; Monkey G: 171.76 ± 97.30 SD trials per day).

#### Composition of lotteries

At each trial, the monkey had to choose between two lotteries. Each lottery *L*_*i*_ with *i* ∈ {1, 2} had two outcomes, *x*_*i*_ and 0. *x*_*i*_ is the outcome of the lottery *i* with probability *p*_*i*_ while 0 is the output of the lottery with probability [1 − *p*_*i*_], with *p*_*i*_ ∈ {0.25, 0.50, 0.75, 1.00}. For all lottery *i*, *x*_*i*_ is a non-zero integer in the interval [−3,3].

We will name thereafter lottery pairs with potential gains LPG (*x*_*i*∈1,2_ > 0), and lottery pairs with potential losses LPL (*x*_*i*∈1,2_ < 0).

Because of a large number of possible lottery pairs, several cases were distinguished and grouped in two sets.

The first set is composed of lottery pairs that were used to evaluate the monkey’s performance. Pairs belonging to this set are those in which there was a stochastic dominant option whatever the attitude towards risk of the subject: *L*_1_ dominates *L*_2_ so the monkey is expected to choose *L*_1_ if it understands the task. The following cases were extracted:

- **Type 1**. *x*_1_ > 0 and *x*_2_ < 0 while *p*_1_ = *p*_2_ (lottery pairs containing one lottery with potential losses and on lottery with potential gains); assess the discrimination of *losses from the gains* (this is the only case included in the design mixing potential gains and potential losses in a single trial); 36 different lottery pairs.
- **Type 2**. *p*_1_ = *p*_2_ and *x*_1_ > *x*_2_, with *x*_*i*∈1,2_ > 0 (LPG with a stochastic dominant option differentiating only by the *x* values); assess the discrimination of *positive quantities*; 12 different lottery pairs.
- **Type 3**. *p*_1_ = *p*_2_ and *x*_1_ < *x*_2_, with *x*_*i*∈1,2_ < 0 (LPL with a stochastic dominant option differentiating only by the *x* values); assess the discrimination of *negative quantities*; 12 different lottery pairs.
- **Type 4**. *p*_1_ > *p*_2_ and *x*_1_ = *x*_2_, with *x*_*i*∈1,2_ > 0 (LPG with a stochastic dominant option differentiating only by the *p* values); assess the discrimination of *probabilities* associated to *positive* quantities; 12 different lottery pairs.
- **Type 5**. *p*_1_ < *p*_2_ and *x*_1_ = *x*_2_, with *x*_*i*∈1,2_ < 0 (LPL with a stochastic dominant option differentiating only by the *p* values); assess the discrimination of *probabilities* associated to *negative* quantities; 18 different lottery pairs.

The second set is composed of lottery pairs that allowed the attitude toward risk of the monkeys to be evaluated, by assessing how the monkey realizes a trade-off between quantity and probability of reward. The following cases were extracted:

- **Type 6**. *p*_1_ < *p*_2_ and *x*_1_ > *x*_2_, with *x*_*i*∈1,2_ > 0 (LPG with no stochastic dominant option); 18 different lottery pairs.
- **Type 7**. *p*_1_ < *p*_2_ and *x*_1_ < *x*_2_, with *x*_*i*∈1,2_ < 0 (LPL with no stochastic dominant option); 18 different lottery pairs.

Indeed, we will consider that when dealing with gains, an option *A* is riskier than an option *B* if *p*_*A*_ < *p*_*B*_ and *x*_*A*_ > *x*_*B*_, that is to say where option *A* leads to a greater amount of reward but with a smaller probability, while option *B* leads to a smaller reward but with a greater probability. We will consider that when dealing with losses, an option *A* is riskier than an option *B* if *p*_*A*_ < *p*_*B*_ and *x*_*A*_ < *x*_*B*_, that is where option *A* leads to a greater loss but with a smaller probability, while option *B* leads to a smaller loss but with a greater probability. Thus, if the subject is risk-seeking, he will be biased toward choosing *L*_1_.

#### Implementation of lotteries, gains and losses

During experimental sessions, the monkeys have restricted access to water five days per week. This motivates them to perform the task. Hence, the potential gain from each lottery was water in place of the monetary gains customarily used in human experiments.

Each trial, the monkey had to choose between two lotteries. A single lottery is represented by a pie chart (see Fig. 2), as proposed by Stauffer *et al*.^11^. Each pie chart is composed of two slices. Starting from a reference point at the top of the circle, the first segment of the pie is shifted by a random angle between 0 and 359°. Each slice encodes one possible outcome of the lottery (*x*_*i*_ or 0). The arc length of each slice represents the probability of this outcome (*p* or [1 − *p*]). The pattern of each slice represents the number of tokens earned or lost. The orientation of the parallel lines constituting the pattern indicates a quantity (see Fig. 2). Hence, horizontal lines represent 0. Rotation of these horizontal lines *clockwise* by one-, two-, three-quarters of 45° respectively represent a loss of −1, −2, −3 tokens. Rotation of these horizontal lines counter-clockwise by one, two, three quarters of 45° represent a gain of 1, 2, 3 tokens.

In each trial, the monkey could gain between −3 and +3 tokens. At the end of the trial, the monkey would be rewarded with water proportional to the tokens gained. Because it is not possible to give negative water, the monkey started each trial with 3 tokens, visible on the screen. Hence, the monkey would have between 0 and 6 tokens at the end of the trial. For each token, the water tank valve would be opened for 150 ms. This duration of opening gave approximately 100 of water. So, at the end of each trial the monkey would receive between 0 and 600 of water.

#### Experimental paradigm

The monkey sat in a primate chair positioned 20 cm from a touch screen. The experimental session was composed of a series of trials. Each trial consisted of several steps (see Fig. 3). At the beginning of the trial, a gauge with 3 tokens was displayed. The monkey had to grasp a grip for a short duration that varied randomly in a range from trial to trial (150~300 ms) to ensure that the monkey could not anticipate the stimuli display.

If the monkey did not hold the grip long enough, the trial was considered as failed and an error was raised. Error trials were concluded with the display of a black screen, and the monkey had to wait 2000ms for the beginning of the next trial.

After the monkey had held the grip for the required amount of time (150~300 ms), stimuli appeared. The stimuli presented were two circles representing two lotteries. The monkey had 2000 ms to decide which lottery to choose. If the monkey did not choose within the allotted decision time, an error was signaled.

After making a choice by touching one of the two circles the other circle disappeared. The monkey then had 5000 ms to return its hand to the grip (otherwise, an error was raised). Once the monkey had returned its hand to the grip the outcome was determined based on the probabilities shown in the two slices of the chosen circle. The amount of reward (loss) was indicated to the animal by the disappearance of the non-selected slice. The gauge filled (emptied) by the amount earned (forfeited), one token at the time. The time of the filling animation was kept constant at 1500 ms. At the end of the trial, the gauge emptied itself and the monkey received one drop of water (100) for each token in the gauge. The time of the emptying animation was kept constant at 1500 ms.

The inter-trial interval varied randomly between 150 and 300 ms.

### Modeling

#### Lotteries, outcomes, and probabilities

Based on prospect theory^3,4^, we constructed a simple decision-making model designed to evaluate the attitudes to risk of the two monkeys. In our task, a lottery *L* has two possible outcomes *x* a non zero integer in the interval [−3, 3] with probability *p* and 0 with probability [1 − *p*]. The formula below supposed the use of a normalized value of *x* such as *x* ∈ [0, 1].

#### Utility functions

We define a function *U* that represents the utility — or the subjective expected value — of a lottery *L*:

$$U({L}_{i})=w({p}_{i})\cdot u({x}_{i})$$

with *w* the probability distortion function and *u* the utility function of an amount *x*_*i*_ ∈ [0, 1]. *w* is defined over the interval [0,1] and is continuous, with *w*(0) = 0 and *w*(1) = 1. *u* is defined over the interval [−1, 1] and is continuous, with *u*(0) = 0 and ∀*x*_*i*_: −1 ≤ *u*(*x*) ≤ 1.

Let us define a utility function *u* such as:

$$u(x)=\{\begin{array}{ll}{u}^{1-{\omega }_{G}} & {\rm{if}}\,x > 0,\\ -|u{|}^{1+{\omega }_{L}} & {\rm{if}}\,x < 0,\\ 0 & {\rm{otherwise}}.\end{array}$$

with *ω*_*G*_ ∈ [−1, 1] the parameter of risk-aversion for gains, and *ω*_*L*_ ∈ [−1, 1] the parameter of risk aversion for losses. Thus, if *ω*_*G*_ is positive, *u*′(*x*) is positive for ∀*x* > 0, meaning an aversion towards risk for gains – this method of modeling attitude towards risk is very common^28^. In the case where *x* is negative and *ω*_*L*_ is positive, *u*′(*x*) is positive ∀*x* < 0, meaning an aversion towards risk in losses.

#### Probability distortion function

We assume that probabilities are perceived in a distorted manner. Following Prelec^29^, we defined a probability distortion function, *w*, in the following manner:

$$w(p)=\exp (\,-\,{[-\mathrm{ln}(p)]}^{\alpha })$$

with *α* = *α*_*G*_ if *x* > 0 and *α* = *α*_*L*_ if *x* < 0. Both *α*-values are drawn from interval (0, 1] and describe to which extent the probabilities are distorted (the closer the *α*-value is to zero, the more the probabilities are distorted).

#### Stochasticity function

We also assume that action selection is probabilistic: the option that is subjectively seen as the best option is chosen only with a greater probability than the other options (and not always). As a classic *softmax* function^30^ to introduce stochasticity can induce non-monotonicity concerning the effect of risk parameter on probability to choose the riskiest option for a given lottery (i.e. monotonicity is not guaranteed for an arbitrary couple of two lotteries), we used the alternative proposed by Apesteguia & Ballester^31^.

Let *L*_1_ be a lottery riskier than *L*_2_ (i.e. *p*_1_ < *p*_2_ and *x*_1_ > *x*_2_). Let *p*(*L*_1_) be the probability of choosing *L*_1_:

$$p({L}_{1})=\frac{\exp (\lambda f({L}_{1},{L}_{2}))}{\exp (\lambda f({L}_{1},{L}_{2}))+\exp (\lambda \omega )}$$

with *ω* = *ω*_*G*_ if *x*_1_ and *x*_2_ are positive and *ω* = *ω*_*L*_ if *x*_1_ and *x*_2_ are negative, *λ* ∈ (0, ∞) a free parameter describing to which extent decision-making is stochastic, and *f* a function that gives the *ω*-value for which the decision-maker would be indifferent between the both lotteries. The lower the *λ*-value is, the more the decision-making is stochastic.

### Analysis

#### Certainty-risk trade-off

We treated separately a singular case similar to one proposed by Kahneman & Tversky’s seminal paper^3^: (i) “In addition to whatever you own, you have been given $1,000. You are now asked to choose between: (A) $1,000 with probability 0.50, and (B) $500 for sure.” and (ii) “In addition to whatever you own, you have been given $2,000. You are now asked to choose between: (C) $ −1,000 with probability 0.50, and (D) $−500 for sure”.

Although we did not vary the initial wealth as Kahneman and Tversky had, we offered a very similar proposition to the monkeys. At the beginning of the trial, they had three tokens and they had the choice between one lottery giving one token (or removing one) for certain, and a second lottery giving two tokens (or removing two) half of the time.

To assess the statistical relevancy of the comparison of the behavior of the monkey depending on whether the lottery pair involved gains or losses, we used a *chi*−*squaredtest*. The null hypothesis was that animals would make an equal number of risky choices in lotteries involving gains and lotteries involving losses. We set the significance threshold at *p* = 0.01.

For Monkey H, we collected 88 trials for the pair of lotteries involving gains, 163 trials for the pair involving losses. For Monkey G, we collected 92 trials for the pair of lotteries involving gains, 144 trials for the pair involving losses.

#### Performance assessing

Lottery pairs with a stochastic dominant option (Type 1–5) were used to verify to check the monkeys’ performance.

Because we did not expect the distribution to be uniform, we computed the median and interquartile range (IQR) of the success rate for each pair of lotteries.

For Monkey H, we collected for lottery pairs Type 1 to 6 the results of respectively 3204, 3290, 6040, 3074, and 3986 trials. For Monkey G, we collected for lottery pairs Type 1 to 6 the results of respectively 2862, 2956, 4969, 2678, and 3473 trials (further details are provided in Table S2).

#### Consideration of the difference of expected values during trade-offs between quantity and probability

Lottery pairs with no stochastic dominant option (Type 6 & 7) were used to verify that the frequency with which the riskiest option was chosen was dependent on the difference between the expected values of the safest option and of the riskiest option.

To assess this relation, we used an ordinary sigmoid function *f* to fit the monkeys’ behavior such as:

$$f({\rm{\Delta }})=1/(1+\exp (\,-\,\beta ({\rm{\Delta }}-\gamma ))$$

with Δ the difference of expected values of the two lotteries, *β* = *β*_*G*_ the slope parameter when considering LPG, and *β* = *β*_*L*_ the slope parameter when considering LPL, *γ* = *γ*_*G*_ the intercept parameter when considering LPG and *γ* = *γ*_*L*_ the intercept parameter when considering LPL. We optimized these two sets of free parameters to fit the data points using a Levenberg-Marquardt algorithm (SciPy library). Margin error have been computed for each parameter value with threshold at *p* = 0.01.

For Monkey H, the number of trials used was 1947 for LPG and 3005 for LPL; for Monkey G, the number of trials was 1794 for LPG and 2641 for LPL (further details are provided in Table S2).

#### Attitude toward risk

In order to characterize this attitude toward risk, we optimized for each monkey separately the free parameters of our model, namely *ω*_*G*_, *ω*_*L*_, *α*_*G*_, *α*_*L*_, *λ*_*G*_, *λ*_*L*_, using a SLSQP optimization algorithm^32^ (Scipy Library). The optimization was based on minimization of the inverse of the log-likelihood for a given set of parameters. Such log-likelihood is computed as follows:

$$\mathrm{ln}\, {\mathcal L} (\theta ;{c}_{1},\ldots ,{c}_{{n}_{{\mathbb{P}}}})=\sum _{i}^{{n}_{{\mathbb{P}}}}\,\mathrm{ln}\,f({c}_{i}|\theta )$$

with *θ* a particular set of values of our five free parameters, ${n}_{{\mathbb{P}}}=168$ the total number of different lottery pairs a monkey encountered, *c*_*i*_ a set of binary values for the pair of lotteries $i\in 1,{n}_{{\mathbb{P}}}$, each of these binary values indicating if the monkey chose the lottery *L*_1_ when facing the pair of lotteries *i* in a particular trial (the size of this set can vary among the lottery pairs depending on the number of trials the pair *i* appears), and *f* being the density function that gives the probability of this observation given the set *θ* of parameters. As each set of choices for a pair of lotteries *i* is composed of binary values (i.e. choosing *L*_1_ or not), let *f* be:

$$f({c}_{i}|\theta )=Pr({k}_{i},{n}_{i},{p}_{i}|\theta )$$

with *n*_*i*_ the number of trials in which the monkey encounters this pair of lotteries *i*, *k*_*i*_ the number of times the monkey chooses *L*_1_ when it encounters the pair of lotteries *i*, and *p*_*i*_, the (theoretical) probability of choosing the lottery *L*_1_ when facing the pair of lotteries *i* given the set of parameters *θ* (naming the output of our model given *θ*). In other words, the value of *f* value depends only on *k*_*i*_, *n*_*i*_ and *p*_*i*_ given *θ*. Knowing that, we can define *f* as the following binomial mass function:

$$f({c}_{i}|\theta )=(\frac{{n}_{i}}{{k}_{i}})[{p}_{i}|{\theta }^{{k}_{i}}]{(1-[{p}_{i}|\theta ])}^{{n}_{i}-{k}_{i}}$$

The trials used for this fit were the same than those for assessing the frequency with which the riskiest option was chosen was dependent on the difference between the expected values of the safest option and of the riskiest option (for Monkey H, the number of trials was 1947 for LPG and 3005 for LPL; for Monkey G, the number of trials was 1794 for LPG and 2641 for LPL; further details are provided in Table S2).

To assess the stability of the fit, we used a cross-validation process by binning the trials in 20 chunks by chronological order for each monkey and computing the average best value over these 20 chunks.

For comparisons of best parameter values for each monkey between those fitting results obtained with LPG and those obtained with LPL, as we did not expect a normal distribution of the data due to clustering effects at the boundaries of the parameter ranges, assessment of statistic relevancy has been made with Mann-Whitney’s U ranking test, applying Bonferroni corrections for multiple comparisons. We set the significance threshold at *p* = 0.01.

## Supplementary information

Supplementary info (PDF)

LaTeX Supplementary File

LaTeX Supplementary File

## Data Availability

The data are available at the same address than the analysis program: https://github.com/AurelienNioche/MonkeyAnalysis.

## Code Availability

The code for monitoring the task and for data analysis has been written in Python, using the Python scientific stack^33–35^. It ran under macOS Sierra. The code for the task is available at https://github.com/AurelienNioche/MonkeyProject. The code for the analysis is available at https://github.com/AurelienNioche/MonkeyAnalysis.
