# Supplementary material for: An asymmetry of treatment between lotteries involving gains and losses in rhesus monkeys
Source: Sci Rep. 2019 Jul 18;9:10441. doi: 10.1038/s41598-019-46975-2 (PMC6639334; doi:10.1038/s41598-019-46975-2)
Supplement: Supplementary file 1 — Supplementary info (PDF) [file 41598_2019_46975_MOESM1_ESM.pdf]

An asymmetry of treatment between lotteries involving gains and losses in rhesus monkeys

Aurélien Nioche[1,2,3,4,5,*] **, Sacha Bourgeois-Gironde**[6,2,3] **, and Thomas Boraud**[4,5,7]
[1]Aalto University, School of Electrical Engineering, Department of Communications and Networking, 02150 Espoo, Finland [2]Institut Jean Nicod, Département d'études cognitives,ENS, EHESS, PSL Research University, 75005 Paris, France [3]Institut Jean Nicod, Département d'études cognitives, CNRS, UMR 8129 [4]Institut des Maladies Neurodégénératives, Université de Bordeaux, 33000 Bordeaux, France [5]Institut des Maladies Neurodégénératives, CNRS, UMR 5293 [6]Laboratoire d'Economie Mathématique et de Microéconomie Appliquée, Université Panthéon Assas, 75006 Paris, France [7]Centre Expert Parkinson, CHU Bordeaux, 33000 Bordeaux, France
[*]Correspondence to: nioche.aurelien@gmail.com

Supplementary

Summary of the different types of lottery pair. Table S1 reports the different types of lottery pairs.

Summary of trial number per type of lottery pair. Table S2 reports the number of trials for each type of lottery pair.

Summary of results for pairs of lotteries with a strictly dominant option. Table S3 reports the median success rates for pairs of lotteries with a strictly dominant option. All interquartile range are above the chance level (0.5).

Summary of the fit of the frequencies of risky choices depending on the difference of the expected values with a 2-parameters sigmoid function. Table S4 reports the best-fit parameter values when fitting the frequency with which the riskiest option is chosen depending on the the difference of expected values with a 2-parameters sigmoid function (one for the slope, β, and one for the intercept, γ).

The results indicate that both monkeys consider the difference of expected values when realizing trade-offs between quantity and probability for lotteries with potential gains (Monkey H: $\beta_G = 1.70\ [0.72, 2.68]$, Monkey G: $\beta_G = 1.83\ [1.55, 2.12]$). However, results indicate also that both monkeys less consider the difference of expected values when dealing with lotteries including potential losses, and we can not reject the hypothesis that they do not consider it at all (Monkey H: $\beta_L = 0.58\ [-0.36, 1.52]$, Monkey G: $\beta_L = 0.45\ [-0.17, 1.07]$).

Summary of the fit of the monkeys' behavior regarding their attitude toward risk with the decision-making model. Table S5 report the means and standard deviations of the best-fit parameter values for the 20 chunks of data. Average log-likelihood sums are respectively -50.38 (± 5.18 SD) and -49.96 (± 5.02 SD) for Monkey H and Monkey G.

Comparison of best-fit parameter values when fitting monkeys' behavior regarding its attitude toward risk with the decision-making model. Table S6 reports the results of the analysis of the statistic relevancy of the comparisons of the best-fit parameter values for each monkey depending of the type of lottery pairs (gains vs losses).

The optimized values of ω_G and ω_L indicate a slight risk-seeking for gains or quasi risk neutrality contrasting with a strong risk-aversion for losses (Monkey H: $\omega_G = 0.28 \pm 0.21$, $\omega_L = -0.71 \pm 0.20$; $u = 0.0$, $p < 0.001$, $n_{obs} = 2 \times 20$; Monkey G: $\omega_G = 0.15 \pm 0.31$, $\omega_L = -0.75 \pm 0.28$); $u = 11.0$, $p < 0.001$, $n_{obs} = 2 \times 20$).

The optimized values of α_G and α_L indicate a slight probability distortion for gains contrasting with a strong probability distortion for losses (Monkey H: $\alpha_G = 0.63$ (± 0.14, $\alpha_L = 0.21 \pm 0.14$; $u = 7.0$, $p < 0.001$, $n_{obs} = 2 \times 20$; Monkey G: $\alpha_G = 0.90 \pm 0.23$), $\alpha_L = 0.30 \pm 0.21$); $u = 24.5$, $p < 0.001$, $n_{obs} = 2 \times 20$).

The comparison of these values the optimized values of λ_G and λ_L reveals no statically difference in stochasticity of choice for gains and losses (Monkey H: $\lambda_G = 2.52 \pm 0.98$, $\lambda_L = 1.82 \pm 0.47$; $u = 104.0$, $p = 0.029$, $n_{obs} = 2 \times 20$; Monkey G: $\lambda_G = 1.71 \pm 0.95$, $\lambda_L = 1.28 \pm 0.66$; $u = 153.0$, $p = 0.625$, $n_{obs} = 2 \times 20$).

Stability of performances for lottery pairs with a stochastic dominant option over time. Figure S1 is similar to Figure 5 but considering separately 20 chunks of data, in order to control for the stability of performances over time.

Stability of best-fit parameter values of the decision-making model over time. In order to assess the stability of best-fit parameter values of the decision-making model over time, for each monkey and for each parameter, we modelled by a linear regression the evolution of the best-fit parameter values for each of the 20 chunk of data. Table S7 reports the results of this analysis. The only statistically relevant result concerns the evolution of ω_L parameter for Monkey H, $\beta = -0.02$, $F = 16.315$, $p = 0.009$, $n_{obs} = 2 \times 20$, showing that Monkey H is slightly more risk-averse for LPL over time.

Figure S2 represents the best-fit parameter values of the decision-making model for the 20 chunk of data over time and the results of the regression analysis. Parameter values represented in this figure are the one used for the construction of Fig. 7, 8, & 9.

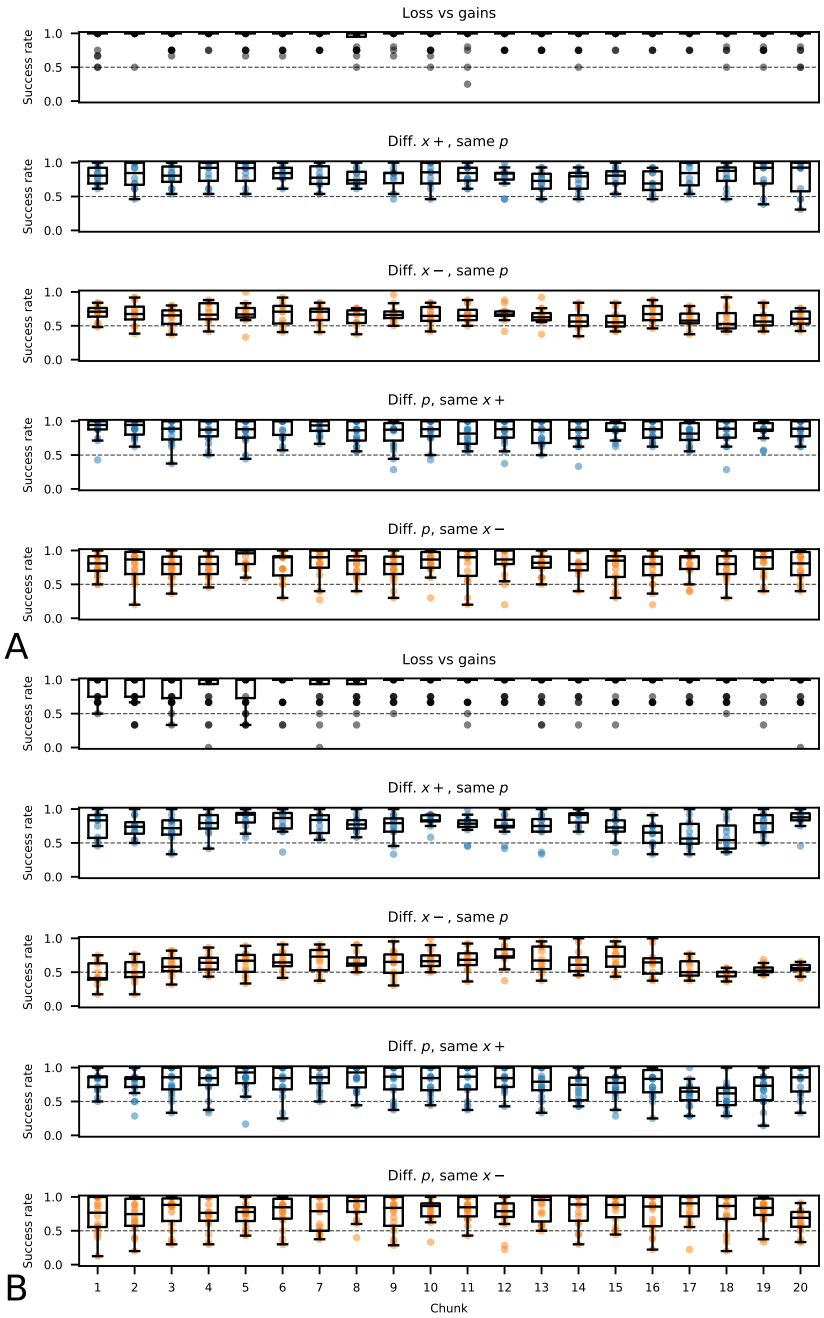

Figure S1. Performances for lottery pairs with a stochastic dominant option over time. Each boxplot represents the performances for a specific chunk of data. Each dot represents the frequency with which the best option was chosen for a particular pair of lotteries (blue: potential gains only; orange: potential losses only) for a specific chunk of data. Each box plot extends from the lower to the upper quartile of frequencies observed, with the central line at the median. The whiskers represent the value of 1.5 IQR. As they are two options, chance level, indicated by a dashed line, is at 0.5. **A.** Monkey H. **B.** Monkey G.

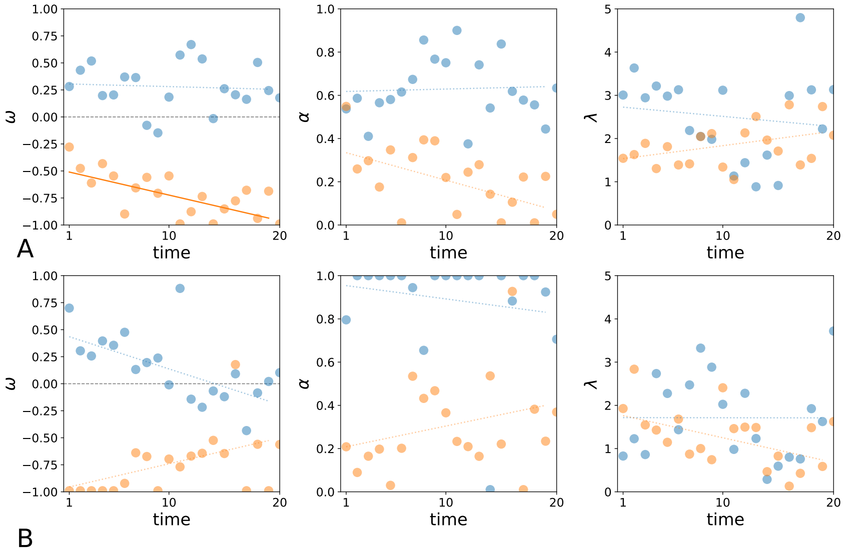

Figure S2. Best-fit parameter values for the decision-making model over time. For each monkey, the best-fit parameter values are shown over time: blue dots for lottery pairs involving gains, orange dots for lottery pairs involving losses. Each dot corresponds to the best value of a specific parameter for a specific chunk of data. The line represents the results of the regression analysis: a plain line indicates a significant result at the 1% level, while a dashed line indicates that the null hypothesis can not be rejected. **A.** Monkey H. **B.** Monkey G.

Pair type	Specificity	n_{pairs}	To assess
1	$x_1 > 0$ and $x_2 < 0$ while $p_1 = p_2$	36	Discrimination of *pos.* from *neg.* x-values
2	$p_1 = p_2$ and $x_1 > x_2$, with $x_{i\in 1,2} > 0$	12	Discrimination of *pos.* x-values
3	$p_1 = p_2$ and $x_1 < x_2$, with $x_{i\in 1,2} < 0$	12	Discrimination of *neg.* x-values
4	$p_1 > p_2$ and $x_1 = x_2$, with $x_{i\in 1,2} > 0$	18	Discrimination of *p*-values with *pos.* x-values
5	$p_1 < p_2$ and $x_1 = x_2$, with $x_{i\in 1,2} < 0$	18	Discrimination of *p*-values with *neg.* x-values
6	$p_1 < p_2$ and $x_1 > x_2$, with $x_{i\in 1,2} > 0$	18	Attitude toward risk with *pos.* x-values
7	$p_1 < p_2$ and $x_1 < x_2$, with $x_{i\in 1,2} < 0$	18	Attitude toward risk with *neg.* x-values

Table S1. Summary of the different types of lottery pair. n_{pairs}: Number of different lottery pairs of a specific type.

Pair type	Related fig.	n^H_{trials}	n^H_{trials} per pair	n^G_{trials}	n^G_{trials} per pair
1	Fig. 5	3204	89.00 ± 10.73 SD	2862	79.50 ± 9.49 SD
2	Fig. 5	3290	274.17 ± 8.36 SD	2956	246.33 ± 11.14 SD
3	Fig. 5	6040	503.33 ± 17.16 SD	4969	414.08 ± 69.72 SD
4	Fig. 5	3074	170.78 ± 14.74 SD	2678	148.78 ± 14.51 SD
5	Fig. 5	3986	221.44 ± 13.54 SD	3473	192.94 ± 21.70 SD
6	Fig. 6, 7, 8, & 9	1947	108.17 ± 12.16 SD	1794	99.67 ± 12.12 SD
7	Fig. 6, 7, 8, & 9	3005	166.94 ± 14.94 SD	2641	146.72 ± 15.29 SD

Table S2. Number of trials depending on the type of lottery pair. n_{pairs}: Number of different lottery pairs of a specific type. n^X_{trials}: Number of trials for monkey X.

Pair type	Monkey H	Monkey G
1	0.98 (IQR $= 0.95 - 1.00$)	0.96 (IQR $= 0.89 - 0.99$)
2	0.85 (IQR $= 0.68 - 0.91$)	0.76 (IQR $= 0.70 - 0.85$)
3	0.62 (IQR $= 0.57 - 0.71$)	0.61 (IQR $= 0.55 - 0.70$)
4	0.86 (IQR $= 0.80 - 0.94$)	0.78 (IQR $= 0.72 - 0.85$)
5	0.87 (IQR $= 0.67 - 0.93$)	0.80 (IQR $= 0.61 - 0.94$)

Table S3. Median success rates for lottery pairs with a strictly dominant option. IQR: Interquartile range.

Parameter	Monkey H	Monkey G
β_G	$1.70\ [0.72, 2.68]$	$1.83\ [1.55, 2.12]$
β_L	$0.58\ [-0.36, 1.52]$	$0.45\ [-0.17, 1.07]$
γ_G	$0.26\ [0.02, 0.51]$	$0.19\ [0.13, 0.25]$
γ_L	$-1.33\ [-3.48, 0.82]$	$-1.63\ [-3.91, 0.64]$

Table S4. Best-fit parameter values when fitting the frequency with which the riskiest option is chosen depending on the the difference of expected values with a 2-parameters sigmoid function . The margin errors are given for each value with threshold at $p = 0.01$.

	ω_G, ω_L	α_G, α_L	λ_G, λ_L
Range	$[-0.99, 0.99]$	$[0.01, 1.00]$	$[0.00, 5.00]$
Monkey H	0.28 (± 0.21); -0.71 (± 0.20)	0.63 (± 0.14); 0.21 (± 0.14)	2.52 (± 0.98); 1.82 (± 0.47)
Monkey G	0.15 (± 0.31); -0.75 (± 0.28)	0.90 (± 0.23); 0.30 (± 0.21)	1.71 (± 0.95); 1.28 (± 0.66)

Table S5. Average of best-fit parameter values when fitting monkeys' behavior regarding its attitude toward risk with the decision-making model. The first row of this table indicates the range for the free parameters. The second and third rows give the average best-fit parameter values ($\pm$SD), respectively for Monkey H and Monkey G. Average log-likelihood sums are respectively -50.38 (± 5.18 SD) and -49.96 (± 5.02 SD) for Monkey H and Monkey G.

Monkey	Comparison	n_{obs}	u	p_{raw}	p_{corr}
Monkey H	ω_G / ω_L	2×20	0.0	$p < 0.001$	$p < 0.001^*$
Monkey H	α_G / α_L	2×20	7.0	$p < 0.001$	$p < 0.001^*$
Monkey H	λ_G / λ_L	2×20	104.0	$p = 0.005$	$p = 0.029$
Monkey G	ω_G / ω_L	2×20	11.0	$p < 0.001$	$p < 0.001^*$
Monkey G	α_G / α_L	2×20	24.5	$p < 0.001$	$p < 0.001^*$
Monkey G	λ_G / λ_L	2×20	153.0	$p = 0.104$	$p = 0.625$

Table S6. Comparison of best-fit parameter values of the decision-making model when fitting monkeys' behavior regarding its attitude toward risk. We compared for each monkey the best-fit parameter values when fitting the results with lottery pairs involving gains and the best-fit parameter values when fitting the results with lottery pairs involving losses. The assessment of statistic relevancy of comparisons of best-fit parameter values has been made with Mann-Whitney's U ranking test, applying Bonferroni's corrections for multiple comparisons. p_{raw} and p_{corr} are p-values respectively before and after Bonferroni correction. '*' indicates that the result of the test is significant at the 1% level. n_{obs} are the number of observations used for computing the value of u, which is the output of the Mann-Withney test.

Monkey	Parameter	n_{obs}	intercept	β	F-statistic	p_{raw}	p_{corr}
Monkey H	ω_G	2×20	0.31	-0.00	0.10	$p = 0.761$	$p = 1.000$
Monkey H	ω_L	2×20	-0.49	-0.02	16.32	$p = 0.001$	$p = 0.009^*$
Monkey H	α_G	2×20	0.62	0.00	0.05	$p = 0.832$	$p = 1.000$
Monkey H	α_L	2×20	0.35	-0.01	8.50	$p = 0.009$	$p = 0.111$
Monkey H	λ_G	2×20	2.75	-0.02	0.36	$p = 0.557$	$p = 1.000$
Monkey H	λ_L	2×20	1.50	0.03	3.65	$p = 0.072$	$p = 0.867$
Monkey G	ω_G	2×20	0.47	-0.03	11.44	$p = 0.003$	$p = 0.040$
Monkey G	ω_L	2×20	-0.98	0.02	5.96	$p = 0.025$	$p = 0.302$
Monkey G	α_G	2×20	0.96	-0.01	0.56	$p = 0.464$	$p = 1.000$
Monkey G	α_L	2×20	0.20	0.01	1.74	$p = 0.204$	$p = 1.000$
Monkey G	λ_G	2×20	1.72	-0.00	0.00	$p = 0.993$	$p = 1.000$
Monkey G	λ_L	2×20	1.82	-0.06	6.06	$p = 0.024$	$p = 0.290$

Table S7. Results of the linear regression analysis of the evolution of the best-fit parameter values of the decision-making model. For each monkey and for each parameter of the decision-making model, we modelled by a linear regression the evolution of the best-fit parameter values for each chunk of data. β is the estimated value of the coefficient of regression. We applied Bonferroni corrections for multiple comparisons. p_raw and p_corr are p values respectively before and after Bonferroni correction. '*' indicates that the result of the test is significant at the 1% level. n_obs are the number of observations used for computing the value of the F-statistic.
